# Case Report: Whining toddler: could it be a serious illness

**DOI:** 10.3389/fendo.2025.1533766

**Published:** 2025-08-01

**Authors:** Sandhya J. Kadam, Archana Reddy Bongurala, Rishika Sharma, Anshuman Srivastava

**Affiliations:** ^1^ Department of Pediatrics, Family HealthCare Network, Visalia, CA, United States; ^2^ Department of Pediatrics, Omni Family Health, Bakersfield, CA, United States

**Keywords:** whining, toddler, type 1 diabetes mellitus, diabetic ketoacidosis, hyperglycemia, anion gap, ketones, lethargy

## Abstract

Children in the developing stages whine for a variety of reasons. It can serve as a way of communicating when they cannot express their needs or feelings verbally. They may whine to seek attention or to deal with discomfort and frustration, especially when they want something but aren’t getting it quickly enough. Additionally, it can be a response to unfamiliar stress, lack of sleep, uncomfortable environment, conflicts with siblings, or separation anxiety. Whining is common in children, usually peaking in toddlers and preschoolers. Depending on how parents respond to the behavior, some children may continue to whine beyond this age. Whining can also occur in elderly patients, especially those with dementia, and may be triggered by physical pain, hunger, psychological distress, or overstimulation in their environment. Whining is usually considered a form of behavior and not a sign of illness. Ignoring it is one option recommended for dealing with it, but rarely, it could be made to signal danger, fear, pain, or some significant underlying illness. However, persistent, unexplained whining shouldn’t be simply ignored. We present a case of Type 1 diabetes mellitus with diabetic ketoacidosis (DKA), where the primary symptom was whining in a toddler who required multiple visits to the clinic and emergency department before a diagnosis was made. Whining was not initially recognized as a severe indicator of illness in this patient. To our knowledge, this is the first reported instance of newly diagnosed Type 1 diabetes mellitus with DKA in a child whose main complaint was whining.

## Introduction

Whining and crying are both vocalizations often associated with emotional distress or unmet needs, particularly in children. These behaviors are categorized as temper tantrums. While the frequency of whining may vary with age, it is characterized by a rising and falling melodic pattern, similar to crying. Both vocalizations may involve high-pitched, monotone, nonverbal sounds (1). Whining is one of the four behaviors that make up the distress component of the Anger-Distress model of tantrums, and it reflects some form of psychological or physical discomfort, as described in Potegal’s 2019 review (2). Tantrums have been observed concerning various medical conditions, with discomfort likely being the common underlying factor. Sokol et al., 2005 have described an alternative perspective on whining in children (3).

Type 1 diabetes mellitus(T1DM) is the most prevalent endocrine disorder affecting children. Diabetic ketoacidosis (DKA) is a common initial presentation, occurring in approximately four-fifths of newly diagnosed pediatric cases. The prevalence of DKA at diagnosis varies across different populations (4).

In children, DKA typically manifests with symptoms such as dehydration, excessive thirst, frequent urination related to hyperglycemia, abdominal pain, and nausea or vomiting. Other signs include a fruity odor on the breath, restlessness, irritability, increased drowsiness, and headaches (5). In some cases, T1DM may present with atypical symptoms, such as flu-like illness, neurologic manifestations (e.g., cerebral edema), musculoskeletal symptoms (e.g., myalgia, weakness), and renal complications (e.g., acute kidney injury due to rhabdomyolysis). Gastrointestinal manifestations, including odynophagia, dysphagia, heartburn, and retrosternal discomfort, may also occur due to esophageal candidiasis (6).

We present a case of new-onset T1DM with DKA that exhibited atypical symptoms. This highlights the importance of maintaining a high index of suspicion when evaluating common behavioral changes in children, as early recognition and prompt management of severe underlying conditions is crucial.

## Case report

A previously healthy, completely immunized 20-month-old male presented for a telemedicine consultation with a two-day history of intermittent whining and a low-grade fever of 100.2°F. The child had also experienced a prolonged sleep episode of 16 hours and three episodes of non-bloody, watery diarrhea. However, by the time of the virtual visit, the child had returned to baseline activity levels and appeared well.

The primary care physician (PCP) reviewed dehydration signs with the mother, advised symptomatic treatment and a bland diet, and encouraged increased fluid intake, especially water and Pedialyte. Return-to-clinic and emergency precautions were provided, and an in-person follow-up was scheduled for the next day.

At an in-person follow-up visit, the mother reported that the child had exhibited increased fussiness, ear tugging, persistent whining, and decreased appetite over the past two to three days. The child maintained adequate fluid intake but decreased food intake. The child’s sleep pattern and bowel and bladder habits remained normal for the past 24 hrs.

On physical examination, the child’s vital signs were stable, and a weight gain of 5.5 ounces was noted compared to the previous visit two months prior. The child displayed persistent whining and separation anxiety. Otoscopic examination revealed an erythematous and bulging right tympanic membrane. A diagnosis of acute otitis media of the right ear was made. The child was initiated on amoxicillin and discharged home with instructions for follow-up and emergency contact information. (Please refer to **Table 1**).

**Table 1:** Vitals.

Vitals	2 months ago	Day of presentation	Change
Height	33.07 cm	33.86 cm	
Weight	24 lbs 2 oz	24 lbs, 7.5 oz	5.5 oz gain
BMI	15.51%	15%	
Heart Rate	112 beats per minute	108 beats per minute	
Respiratory rate	28 per minute	30 per minute	
Temperature	98.2 F	97.7 F	

Six days later, the patient returned to the clinic with a five- to six-day history of excessive sleep, averaging 16–18 hours per day. Notably, he was passing urine and stool appropriately. At the last visit, when the diagnosis of otitis media was made, the mother was told that the whining could be related to the discomfort and pain associated with the ear infection. However, during this visit, ear tugging and otitis media were resolved, and all other symptoms had improved, the child continued to exhibit persistent whining and excessive sleeping. Given these concerning symptoms, the primary care physician contacted the emergency department with their concern, and the patient was referred to the emergency department for further evaluation and workup.

The emergency department physician contacted the PCP to report negative COVID-19 and influenza results and to inform them that the patient was discharged to follow up with the PCP as upon presentation, the child was initially tearful and apprehensive but quickly calmed down, engaged in play, drank five juice boxes, and returned to normal behavior. A thorough physical and neurologic examination showed no significant abnormalities, such as petechiae, signs of sepsis, pneumonia, meningitis, myocarditis, or other severe infections. The mother was reassured and felt comfortable managing the child at home, so no further diagnostic testing was necessary.

At follow-up with PCP, the mother reported improvement in the child’s appetite, activity level, and social interaction. Although persistent whining continued, while other symptoms fluctuated, the child’s behavior during the emergency department visit, such as increased fluid intake and improved mood, called into question the severity of the illness. The mother reported that after the reassurance at the emergency department, she is taking whining as just a common behavior for the patient’s age, dealing with it positively, and elected to continue monitoring the child at home and seek further medical attention if needed.

Four days later, the child presented to the emergency department with lethargy, polyuria, and emesis. The mother reported a marked change in the child’s behavior, including excessive sleep, decreased appetite, and increased urinary output. (Please refer to **Table 2**).

**Table 2:** Lab findings.

Urine Analysis	Lab results	Normal Value
UA Clarity	Clear	Clear
UA Color: Yellow	Yellow	Yellow
UA pH: 5.0	5	7
UA Specific Gravity: 1.020	1.020	1.010-1.030
UA Protein: Negative	Negative	Negative
UA Glucose:	>= 1000 (HIGH)	Negative
UA Ketones:	>= 160 (HIGH)	Negative
UA Leuk Esterase	Negative	Negative

Laboratory evaluation demonstrated significant hyperglycemia, metabolic acidosis, and ketonuria, consistent with diabetic ketoacidosis (DKA). The emergency department consulted with the pediatric intensive care unit at Children’s Hospital for further management.

Following recommendations from the pediatric intensive care unit, the patient received intravenous fluid boluses and an initial dose of regular insulin. Blood glucose monitoring was initiated. After fluid resuscitation, the patient’s clinical status and vital signs improved. There were no acute neurologic changes, and the risk of cerebral edema remained low. The patient was subsequently transferred to the Children’s Hospital for further management. (Please refer to **Table 3**).

**Table 3:** Lab findings.

Blood Test	Lab Results	Normal Range
PH:	7.25	7.35-7.45
PCO2	14.8	26–41 mm/Hg
PO2	162.1	25–40 mm/Hg
Bicarbonate	6.4	17–25 mmol/L
Total CO2	6.9	23–29 mEq/L
O2 Saturation	99	94-100%
Sodium:	129	135–145 mEq/L
Potassium:	4.8	3.4-4.7 mEq/L
Glucose:	450	60–180 mg/dl
Anion Gap	32.8	8–19 mmol/L
BUN	18	7–20 mg/dl
Chloride	94	90–110 mEq/L
Beta-hydroxybutyrate	11.22	0.040 mmol/L

The patient was discharged three days later with a prescribed insulin regimen and blood glucose monitoring before meals, at bedtime, and 2 AM. The family received diabetes education. However, the patient’s father measured a blood glucose level of 540 mg/dL on the evening of discharge. Unable to reach the endocrinologist, the family returned to the hospital. Point-of-care testing in the emergency department confirmed hyperglycemia. The patient received insulin therapy and was readmitted for hyperglycemic management, although DKA was not present. (Please refer to **Table 4**).

**Table 4:** Lab findings.

Blood Test	Lab Results	Normal Range
Sodium	130	135–145 mEq/L
CO2	18	23–29 mEq/L
Anion Gap	20.6	8–19 mmol/L
Glucose level	388	60–180 mg/dl
BUN	28	7–20 mg/dl
BUN/Creatinine	41	3-14
Creatinine	0.68	0.3-0.7
Beta-hydroxybutyrate	5.75	0.040 mmol/L
PH venous	7.35	7.31-7.41
PCO2	35	26–41 mm/Hg
PO2	30	26–41 mm/Hg
Bicarbonate	19.3	17–25 mmol/L

The long-term management of Type 1 DM in the patient was complicated by the challenges of management in a young diabetic patient. Insulin dosing errors were identified as contributing to the patient’s recent hyperglycemic episode, prompting further parental education. Two months later, the patient was readmitted with the presentation of DKA. During this hospitalization, the family received comprehensive dietary counseling. Thyroid function and antibody tests were also performed and were negative. Ongoing follow-up with the primary care physician and endocrinologist continued. Around age four, the patient began insulin pump therapy and is now well-managed, with a recent HbA1c of 7%, improved from 13 at the time of diagnosis.

## Discussion

T1DM is one of the most common chronic conditions in children, resulting from insulin deficiency caused by autoimmune destruction of the insulin-producing beta cells in the pancreas. In the United States, the majority of children with new-onset T1DM present with the classic signs and symptoms of hyperglycemia, and approximately one-third present with DKA. Neurological manifestations are rare (7).

Even though most new cases of T1DM and DKA present with classic symptoms, a few reported cases present with atypical symptoms, as seen in our patient.

Five cases of new-onset T1DM in children, each with unusual neurologic manifestations, including cerebellar ataxia, foot drop, hemiballismus-chorea, reversible peripheral neuropathy, and stroke, are reported (7). These cases highlight the diverse atypical neurologic presentations in pediatric T1DM.

Hyperosmolar hyperglycemic state (HHS) is a severe but rare hyperglycemic crisis in children with diabetes, posing significant morbidity and mortality risks. Early recognition and management are critical for better outcomes. A rare case of HHS as the initial manifestation of T1DM is reported in a 7-year-old girl (8).

A case of Cushing syndrome with DKA is reported as an unusual presentation in an adolescent patient. She initially presented with amenorrhea, oral moniliasis, acanthosis nigricans, moon facies, central obesity, and striae. Given the presence of acanthosis nigricans, urine and blood sugar tests were conducted, revealing hyperglycemia, ketonuria, and glucosuria. Although she did not have the typical symptoms of DKA, the combination of hyperglycemia, ketonuria, and glucosuria prompted an arterial blood gas test, which revealed metabolic acidosis. Consequently, she was diagnosed with DKA (9).

An atypical case of new-onset T1DM in a patient with no prior medical history or family history of autoimmune diseases is reported. She initially presented with a new-onset, painless left foot drop. Blood tests revealed elevated blood sugar and ketone levels, leading to a diagnosis of T1DM. After eight weeks of insulin therapy, her foot drop significantly improved. The foot drop was determined to be related to diabetic neuropathy, which was the presenting symptom of her T1DM (10).

A 16-year-old female patient presented with blurry vision and was found to have bilateral cataracts. She was ultimately diagnosed with T1DM and DKA, and her cataracts were attributed to diabetic cataracts (11).

Symptoms of DKA can progress rapidly, often within 24 hours, and may be more challenging to identify in young children who are still in diapers and are nonverbal. Our patient presented with atypical symptoms of DKA and T1DM. A heightened clinical suspicion for persistent whining, combined with a simple point-of-care glucose measurement, could have led to an earlier diagnosis of diabetes and potentially prevented the development of more severe complications in this patient.

## Conclusion

Whining, a common behavioral expression in young children, typically resolves spontaneously with age and simple interventions. However, persistent whining can sometimes signal an underlying medical condition. While most cases of newly diagnosed T1DM and diabetic DKA present with classic symptoms, atypical presentations may occur. This case report underscores the importance of recognizing persistent whining in children as a potential indicator of severe underlying medical conditions, such as T1DM and DKA.

During the early stages of the presentation, the mother found it unusual that the patient, typically a happy and smiling child, was persistently whining, a behavior not previously seen in him or any of his siblings. Despite her concern, she remained calm and cooperative with healthcare professionals throughout the diagnostic process and continued to be supportive even after the diagnosis.

At the most recent visit, she expressed satisfaction with the patient’s progress, noting that he is doing well on an insulin pump and consistently following up with an endocrinologist and a primary care physician. She is particularly pleased with the patient’s well-controlled HbA1c levels.

## Data Availability

The original contributions presented in the study are included in the article/**Supplementary Material**. Further inquiries can be directed to the corresponding author.
